# Splicing Factor Mutations and Disease Phenotype: Searching for a Needle in a Haystack

**DOI:** 10.1097/HS9.0000000000000587

**Published:** 2021-06-01

**Authors:** Kevin Rouault-Pierre

**Affiliations:** Centre for Haemato-Oncology, Barts Cancer Institute, Queen Mary University of London, United Kingdom

T he democratization of genomic and transcriptional profiling has revealed widespread mRNA splicing alterations in cancer,^1–4^ responsible for dysfunctional gene splicing that can affect disease initiation, propagation, and treatment response.^5^ For instance, in myeloid leukemia including clonal hematopoiesis, splicing factor mutations are initiating events acquired in the most immature hematopoietic compartment, whereas in chronic lymphoid leukemia or breast cancer, mutations in splicing factors are considered secondary hits that contribute to drug treatment resistance.

In clonal hematopoiesis, myeloproliferative neoplasms, acute myeloid leukemia and myelodysplastic syndrome (MDS), core spliceosomal factors are recurrently mutated, and at least 1 out of 2 MDS patients harbor mutations in a splicing factor.^6–8^ Most introns are spliced by the major spliceosome and only less than 1% of human introns are spliced by the minor spliceosome. Mutations in the major (U2-type introns) and minor (U12-type introns) spliceosomes include mutations in 4 core spliceosomal factors: *SF3B1, SRSF2,* the small subunit of the U2AF heterodimer *U2AF1*, and the component of minor spliceosome, *ZRSR2*.

One of the major challenges with understanding the role of splicing factors in causing disease is to discern, among the hundreds of misspliced transcripts observed, which are responsible for the disease phenotype.

While *SF3B1*, *SRSF2*, and *U2AF1* are essential to the major spliceosome, *ZRSR2* is the only 1 out of the 4 to be mainly involved in the minor spliceosome functions. Inoue et al^9^ exploited this feature of *ZRSR2* to shed light on the role of minor intron retentions in driving clonal expansion and disease propagation.

The authors generated mice models with conditional knock out of *Zrsr2* restricted to the hematopoietic compartment and surprisingly, contrary to previous models that evaluated the effect of hotspot mutations in splicing factors of the major spliceosome, the loss of *Zrsr2* promoted hematopoietic stem cell (HSC) self-renewal. *Zrsr2*-null HSCs showed enhanced clonogenic capacities in vitro and out-competed *Zrsr2* WT cells in vivo. Using eCLIP-sequencing to map RNA binding targets and RNA-sequencing from The Cancer Genome Atlas (TCGA) they showed that only a third of U12-type introns are sensitive to *ZRSR2* loss and that these introns are characterized by a 3′ splice site-proximal adenosine branch point that closely resembles the U12-snRNA consensus with a weak or absent polypyrimidine tract. Subsequently a functional genomics screen revealed that *LZTR1*, a cullin-3 adaptor regulating ubiquitin-mediated suppression of RAS-related GTPases, is the target of the minor spliceosome. Indeed, loss of ZRSR2 impairs *LZTR1* minor intron excision and promotes clonal expansion. Interestingly, loss of function of LZTR1 was previously reported in glioblastoma, schwannomatosis and in Noonan Syndrome, a RASopathy. The authors confirmed that *LZTR1*’s loss of expression happens through activation of the nonsense mRNA decay, in MDS-ZRSR2 mutant bone marrow cells. They also showed in one pedigree with autosomal recessive Noonan syndrome that *LZTR1*’s mutation occurs in the branch point region of the minor intron and induces a loss of protein expression. Finally, using TCGA they investigated *LTZR1*’s minor intron splicing across cancers and of note observed alternative splicing of *LTZR1* in a substantial number of tumors, even though components of the minor spliceosome were not mutated.

These findings promote a general acceptance that aberrant splicing is a pan-cancer hallmark driving disease progression. It also highlights the benefit of using HSCs and MDS models to characterize splicing factors’ impact on disease propagation not only in hematological malignancies but also across cancers. It is interesting to observe that despite conferring a clear clonal advantage, ZRSR2 is the least frequently mutated of the 4 core spliceosomal factors (SF3B1, SRSF2, U2AF1, and ZRSR2) in MDS. Furthermore, it was intriguing to see that loss of *Zrsr2* could rescue impaired clonogenic capacities of *Sf3b1* mutant mice, which is in strong contrast with the lethal phenotype of combined *Sf3b1* and *Srsf2* mutant mice. Indeed, it is rare to identify more than one RNA splicing factor mutation in individual patients.^10^

As our journey in unveiling key splicing events progresses, many questions arise. Minor introns are highly conserved in evolution; however, it is less the case for major introns and mice models struggle to recapitulate key splicing events identified in primary human samples, slowing down our understanding of the disease’s cause. This observation is likely to be due to ribonucleic sequence discrepancies between human and mouse. Besides, splicing events are dependent on the expression of variants, which might vary between lineages and tissues, showing different phenotypes during differentiation. Likewise, it is probable that cumulative missplicing events are necessary to reveal an overt disease phenotype.

Progress in genomics and transcriptomics has revealed widespread mRNA splicing alterations in cancers. We are just at the dawn of understanding how mutations in splicing factors, RNA binding protein or intronic regions can induce alternative splicing with dramatic impacts on cell biology.

## Disclosures

The authors have no conflicts of interest to disclose.

## References

[1] Wang GS Cooper TA. Splicing in disease: disruption of the splicing code and the decoding machinery. Nat Rev Genet. 2007; 8:749–761. 17726481 10.1038/nrg2164

[2] Anande G Deshpande NP Mareschal S. RNA splicing alterations induce a cellular stress response associated with poor prognosis in acute myeloid leukemia. Clin Cancer Res. 2020; 26:3597–3607. 32122925 10.1158/1078-0432.CCR-20-0184

[3] Escobar-Hoyos L Knorr K Abdel-Wahab O. Aberrant RNA splicing in cancer. Annu Rev Cancer Biol. 2019; 3:167–185. 32864546 10.1146/annurev-cancerbio-030617-050407 PMC7453310

[4] Wang E Aifantis I. RNA splicing and cancer. Trends Cancer. 2020; 6:631–644. 32434734 10.1016/j.trecan.2020.04.011

[5] Rahman MA Krainer AR Abdel-Wahab O. SnapShot: splicing alterations in cancer. Cell. 2020; 180:208–208.e1. 31951519 10.1016/j.cell.2019.12.011 PMC7291876

[6] Papaemmanuil E Gerstung M Bullinger L. Genomic classification and prognosis in acute myeloid leukemia. N Engl J Med. 2016; 374:2209–2221. 27276561 10.1056/NEJMoa1516192 PMC4979995

[7] Pellagatti A Armstrong RN Steeples V. Impact of spliceosome mutations on RNA splicing in myelodysplasia: dysregulated genes/pathways and clinical associations. Blood. 2018; 132:1225–1240. 29930011 10.1182/blood-2018-04-843771 PMC6172604

[8] Yoshida K Sanada M Shiraishi Y. Frequent pathway mutations of splicing machinery in myelodysplasia. Nature. 2011; 478:64–69. 21909114 10.1038/nature10496

[9] Inoue D Polaski JT Taylor J. Minor intron retention drives clonal hematopoietic disorders and diverse cancer predisposition. Nat Genet. 2021 April 12. [Epub ahead of print]. 10.1038/s41588-021-00828-9 PMC8177065 33846634

[10] Taylor J Mi X North K. Single-cell genomics reveals the genetic and molecular bases for escape from mutational epistasis in myeloid neoplasms. Blood. 2020; 136:1477–1486. 32640014 10.1182/blood.2020006868 PMC7515689

